# Supplementary material for: “In an ideal world that would be a multiagency service because you need everybody’s expertise.” Managing hoarding disorder: A qualitative investigation of existing procedures and practices
Source: PLoS One. 2023 Mar 9;18(3):e0282365. doi: 10.1371/journal.pone.0282365 (PMC9997939; doi:10.1371/journal.pone.0282365)
Supplement: S1 Text — (DOCX) [file pone.0282365.s001.docx]

**Focus Groups Topic Guide**

What does hoarding mean to you?

- - Definition
  - Maintaining factors
    - What does hoarding achieve for the person?
    - How do the people in relationships with the hoarder play a part (simple tolerance - actively taking part)?
    - What role do wider systems have in maintaining the hoarding behaviour (sometime interventions can be counter-productive)?
    - Do environmental factors play a role for example, the balance of space available to stuff hoarded?

Experience of people who hoard/hoarding

- - Numbers?
  - Case studies?
  - Typical characteristics?
  - Outcomes?

Identification of hoarding

- - How have people who hoard to excess been identified?
    - Tenancy visits?
    - Repair visit/Gas service?
    - Complaint from neighbours?
    - External agency contact?
    - Fire Service initiatives?
    - Other?
  - Screening tools used?
  - Safeguarding policy?

Case resolution

- - Policy?
  - Clearance?
  - Support?
    - Family?
    - Health care?
    - Therapy?
  - Capacity?
  - Enforcement?
  - Involvement of other agencies?
