# Supplementary material for: “In an ideal world that would be a multiagency service because you need everybody’s expertise.” Managing hoarding disorder: A qualitative investigation of existing procedures and practices
Source: PLoS One. 2023 Mar 9;18(3):e0282365. doi: 10.1371/journal.pone.0282365 (PMC9997939; doi:10.1371/journal.pone.0282365)
Supplement: Attachment — Submitted filename: Response to reviewer.docx [file pone.0282365.s002.docx]

Reviewer #1: “In an ideal world that would be a multiagency service because you need everybody’s expertise.” Best practice for managing hoarding disorder: A qualitative investigation of existing procedures and practices. The study presents the attitudes and practices of key intervenors in case management of hoarding behaviour. Authors point out that the management of hoarding behaviour in the community is difficult, that attitudes and practices of stakeholders are homogeneous, and that creation of the multiprofessional team might be difficult. It is a timely and needed study.

**Thank you for recognising the importance of our study. We appreciate your constructive feedback and have amended our manuscript in light of these comments. We hope you find the manuscript further improved and therefore now suitable for publication. Below is a point by point response to your comments.**

Its only limit is that it omits the wide international experience in creating multi-professional teams for hoarding disorder around the world. An extensive body of academic and gray literature exists on the topic. Integrating this literature into the introduction and discussion would greatly improve the value of the current work.

**Thank you for this comment. We were not aware of the body of published and grey literature on multi-agency teams for hoarding disorder, particularly the various Canadian initiatives. Based on the information you provided and via forward and backward citation searching we feel that we have now identified this literature, and this has now been added to the manuscript strengthening both the introduction and discussion.**

In addition, the reading of the paper by a clinical psychologist is desirable in those which concern the distinction between hoarding behaviour and hoarding disorder, as well as more ancient and more recent cognitive models of hoarding disorder.

**One of the authors (RC) is a clinical psychologist and she has carefully checked the paper particularly in terms of terminology and cognitive models of hoarding disorder. The manuscript relates to hoarding disorder (rather than behaviour) therefore we have amended terminology accordingly throughout the manuscript. Models of hoarding disorder have also been added.**

Title and abstract.

“In an ideal world that would be a multiagency service because you need everybody’s expertise.” Best practice for managing hoarding disorder: A qualitative investigation of existing procedures and practices. Short Title: Best practice for managing hoarding disorder – The title is incorrect. The term “best” practice is the. I would suggest avoiding the term “best practice” as misleading: it evokes clinical guidelines and systematic reviews.

**We have removed all reference to “best practice” (in both title, short title and throughout the manuscript) as suggested so as not to mislead the reader.**

Introduction.

LL 35-36. “’ Possessions are accumulated over time and many of these items are given sentimental value”. See reasons for hoarding in Moulding, Knight, O'Connor. Also, see the cognitive model of hoarding in Frost and Hartl.

**Reasons for accumulating possessions have been clarified and referenced along with suggested reasons for hoarding disorder with references (Moulding R, Kings C, Knight T. The things that make us: self and object attachment in hoarding and compulsive buying-shopping disorder, Current Opinion in Psychology. 2021; 39: 100-104/Frost RO, Hartl TL. A cognitive-behavioral model of compulsive hoarding. Behaviour Research and Therapy. 1996; 34 (4): 341-350)**

LL 36-37. “such behaviour may be adaptive by ensuring survival when resources become scare”. Interesting tackle, but still unapproved. Sources are needed

**The source of this theory has been added. (Grisham JR, Barlow DH. Compulsive Hoarding: Current Research and Theory. Journal of Psychopathology and Behavioral Assessment. 2005; 27(1): 45-52)**

LL 38-39 However, in a minority of cases the normal hoarding tendency becomes pathological, and the person hoards uncontrollably. See Nordsletten for the exact proportion of problematic hoarding in overall hoarding behavior.

**We have added and referenced the prevalence of hoarding disorder within this sentence using both the Nordsletten citation as suggested (Nordsletten A, Reichenberg A, Hatch S, De la Cruz L, Pertusa A, Hotopf M, Mataix-Cols D. Epidemiology of hoarding disorder. British Journal of Psychiatry. 2013; 203(6): 445-452.) and a more recent systematic review (Postlethwaite A, Kellett S, Mataix-Cols D. Prevalence of hoarding disorder: A systematic review and meta-analysis. Journal of Affective Disorders. 2019; 256: 309-316.)**

LL 40-42 “Hoarding behaviours are characterised by: the acquisition of, and failure 40 to discard, a large number of items that are of limited value; significant clutter in living spaces that render the activities associated with those spaces very difficult; and significant distress or impairment in functioning caused by the hoarding behaviours”. The clear distinction between “hoarding behaviour” and “hoarding disorder” is to discuss

**As previously stated, the manuscript relates to hoarding disorder (rather than behaviour) therefore we have amended terminology accordingly throughout the manuscript.**

LL 43 and further. The term “hoarding” is to avoid. Instead, use either “hoarding behaviour” or “hoarding disorder”, depending on what you mean.

**Thank you for identifying these inconsistencies in the terminology we have used in the manuscript. As previously stated, this manuscript relates to hoarding disorder, and we have amended the manuscript where possible to avoid use of the term “hoarding”. Please note that we have not made any amendments to participants direct quotations as these have been transcribed verbatim.**

LL 48-52 “Currently there is little information about people who hoard from normative community samples, as such individuals rarely come to the attention of research teams but estimates of its prevalence range from 1.5-6% [5] with a recent systematic review concluding that approximately 2 in every 100 people in the general population meet the criteria for hoarding disorder [6]”. For the prevalence of hoarding behaviour see Chaplin (4%). For the prevalence of hoarding behaviour AND hoarding disorder, see Nordsletten

**As previously stated, this manuscript relates to hoarding disorder. We have added and referenced the prevalence of hoarding disorder within the sentence on line 38-39 as suggested using the Nordsletten citation (Nordsletten A, Reichenberg A, Hatch S, De la Cruz L, Pertusa A, Hotopf M, Mataix-Cols D. Epidemiology of hoarding disorder. British Journal of Psychiatry. 2013; 203(6): 445-452.) and a more recent systematic review (Postlethwaite A, Kellett S, Mataix-Cols D. Prevalence of hoarding disorder: A systematic review and meta-analysis. Journal of Affective Disorders. 2019; 256: 309-316.). This sentence (line 48-52) has therefore been deleted.**

LL 53 “Hoarding is a social [7], economic [8-9] and public health problem [10-11] and people who hoard experience a significant reduction in quality of life [12]” First, the references are too old and contain general information on the burden of hoarding. See further works on the hoarding behaviour and risks of fire hazards, evictions, family relationships etc.

**Thank you for this suggestion. We have used more recent citations to support this sentence where necessary and have added some more specific consequences of hoarding disorder.**

LL 54-56 Complaints of hoarding are addressed by multiple community services who have their own procedures and practices in relation to hoarding. In supported housing for example, people who hoard create a series of challenges relating to health and safety, risk management, and safeguarding [13] See publications on the work of multi-professional teams in Laurentides, Quebec, Montreal, Quebec, and further, as well as hoarding task forces across the USA to put your work in the context of existing initiatives.

**Thank you for this comment. We were not aware of the body of published and grey literature on multi-agency teams for hoarding disorder, particularly the various Canadian initiatives. Based on the information you provided and via forward and backward citation searching we feel that we have now identified this literature, and this has now been added to the manuscript in both the introduction and discussion in order to put our work in the context of existing initiatives.**

LL 57-59 In order to aid the development of a possible intervention for hoarding behaviours we aimed to identify current best practice by investigating key stakeholders’ existing practice with regard to identification, assessment and intervention associated with people who hoard. See the survey of primary mental health services conducted in Quebec, Canada, to put your work in the context of existing initiatives. The "best practices" is a term to avoid.

**We have removed all references to “best practice” (in both title, short title and throughout the manuscript) as suggested so as not to mislead the reader. We have also added additional references, particularly in the discussion, to place our work in the context of existing initiatives including the survey of primary mental health services in Quebec, Canada.**

LL 63-64. We believe that, in accordance with Normalisation Process Theory [14], new interventions have the best chance of succeeding if they are based on an awareness and active engagement with existing organisational culture and practices. As you refer to the Normalisation Process Theory, it should be presented, at least in scratch.

**Thank you for this suggestion. A brief, basic description of Normalisation Process Theory has been added to the manuscript.**

Materials and Methods

LL 72 “stakeholders”. The term is to explain shortly here. International readers might misunderstand it.

**Apologies for this oversight, we have added a brief explanation of the term stakeholders.**

Table 1. The absence of building security and fire protection professionals among stakeholders is surprising. The police services seem to be underrepresented as well. To discuss in limits.

**Both housing and fire service professionals were well represented on our focus groups, but we agree that the police services were underrepresented however this has already been reported as a limitation.**

Discussion

The discussion mentions only one study out of many, discussing multi-professional initiatives in hoarding disorder. Large Canadian initiatives, already institutionalized, are overlooked (only in Canada: Saint-Jerome, Montreal, Vancouver, Edmonton).

**Thank you for this comment. As mentioned previously we were not aware of the body of published and grey literature on multi-agency teams for hoarding disorder, particularly the various Canadian initiatives. Based on the information you provided and via forward and backward citation searching we feel that we have now identified this literature, and this has now been added to the manuscript in both the introduction and discussion in order to put our work in the context of existing initiatives.**
