# Supplementary material for: “In an ideal world that would be a multiagency service because you need everybody’s expertise.” Managing hoarding disorder: A qualitative investigation of existing procedures and practices
Source: PLoS One. 2023 Mar 9;18(3):e0282365. doi: 10.1371/journal.pone.0282365 (PMC9997939; doi:10.1371/journal.pone.0282365)
Supplement: Attachment — Submitted filename: Second response to reviewers.docx [file pone.0282365.s003.docx]

**Reviewer #1: The work is much improved. There are some minor corrections left. Thank you very much for the efforts and time you invested in it. *HD - hoarding disorder.**

We are pleased that you think the manuscript is improved and thank you for suggesting the further minor corrections which we have made. We hope you find the manuscript now suitable for publication.

**L21-23. There is an APA definition of HD; there is a discussion on the cut-off point dividing the clinical and non-clinical populations.**

Thank you, we have now changed the terminology in relation to our results L21-23 from “definition” to “understanding” and “prevalence” to “number of cases” so that there is no confusion with the APA definition of hoarding disorder and clinical prevalence.

**L35 – The term "feasibility" is to avoid, it has its reserved maining in health studies.**

Thank you for this comment. We understand your concern and have therefore removed any reference to the term “feasibility”.

**L 38-48. Rewrite. The idea is good; the text is difficult to understand.**

We have rewritten this paragraph and hope that it is now easier to understand.

**L 49: “limited value” is to redefine. At least, you may put the word in the quotes. The processions are valuable for people with HD.**

You are correct in highlighting that the possessions are considered valuable to people with hoarding disorder. Therefore, we have amended the phrase “of limited value” to “regardless of their actual value” in line with the DSM definition of hoarding disorder. This phrase has been amended in both the abstract and introduction.

**L 51 The distress is caused by the need to get rid of processions. Possessions themselves are the source of emotional security.**

Again, you are correct in highlighting that distress is caused by the need to get rid of possessions. Therefore, we have added that there is “a perceived need to save the items and distress associated with discarding them” in line with the DSM definition of hoarding disorder. This phrase has been amended in both the abstract and introduction. We have however retained the phrase “causing significant distress or impairment in functioning” as this is also in line with the DSM definition which states “hoarding causes clinically significant distress or impairment in social, occupational, or other important areas of functioning”.

**L52-58 I see no need for this phrase. It destroys the logic of your narration.**

Thank you for this suggestion. We have deleted this sentence.

**L70-73: the word "community" is used two times in the same phrase.**

Thank you for identifying this repetition. We have deleted “in the community at the end of this sentence”.

**L 83-85. The paragraph on the NTP is not clear enough. Some further explications are needed.**

Apologies if this paragraph was not clear, we have added some further explanation of NPT.

**L 96 professional interest?**

This is helpful, thank you, we have added that key stakeholders are people with a **professional** interest or concern in hoarding disorder.

**Results**

**Results: prevalence term has its definition and is to avoid in your context. It would be better to use "number of cases in professional practice", or "number of referrals".**

Thank you for this suggestion, we have changed the term “prevalence” to “number of cases”.

**In your results, you are working with the part of the reality presented in your introduction. As such, high attention to HD caused by TV shows should be mentioned in the introduction first. For references, see Tolin and Frost.**

Thank you for this comment, we have mentioned in the introduction the increased public awareness of hoarding disorder because of TV shows with appropriate references.

**Discussion**

**I would avoid the "definition of HD" as we already have one in the DSM-V. It may be said as "what is the level of the HD severity demanding intervention from …. (name of services).”**

Thank you, we have now changed the terminology used throughout the manuscript from “definition” to “understanding” and “prevalence” to “number of cases” so that there is no confusion with the APA/DSM-V definition of hoarding disorder and clinical prevalence.

**Ottawa's report "No room to spare" may give insight on the "ideal" organization of care for HD, as far as we are talking about the ideal world.**

Thank you for this suggestion, we have added some details from the “no room to spare” and another similar report in the conclusion section giving insight into the “ideal” organisation of care for hoarding disorder.

**Reviewer #2: Thank you for the invitation to review this revised manuscript on community-based interventions for hoarding. The manuscript presents a qualitative study of two focus groups comprised of 17 professionals from mental health professions, housing, fire prevention, law, and protective services. Understanding more about community-based interventions for hoarding is of great societal importance. The relevance/centrality of normalization process theory to this study was unclear. The definition given on p. 5 sounds good, but how the theory informed the data collection, analysis, or interpretation was not apparent.**

Thank you for this comment. NPT states that new interventions have the best chance of succeeding if they are based on an awareness and active engagement with existing organisational culture and practices therefore NPT influenced data collection methods, topic guide and data analysis. This has been added to the text.

**Lines 83-84 seem to suggest the importance of pilot-testing an intervention after articulating the specific points of intervention – those steps seem to have already been taken by some of the community partnerships, task forces, and response models cited in lines 63-65.**

The complex intervention that has been developed from this study is a psychology led multiagency model which, although having some similarities to other community partnerships, community response models, community task forces and collaborative agreements, is novel in that it is psychology led and therefore would need pilot testing. The novel nature of our complex intervention has been highlighted in the conclusion section. In addition, community partnerships, community response models, community task forces and collaborative agreements have not been translated into either UK policy or practice and therefore would need to be piloted in this context (with a very different health and social care system). We have clarified this further in the introduction.

**I did not review the original submission, but I can see that many citations have been added to reflect some of the published and grey literature on multidisciplinary intervention teams in other countries. The discussion of those other initiatives and research programs is helpful, but it also adds confusion about the value of the present study. Overall, the rationale and conceptual foundation for the study is unclear. The manuscript points to numerous community-based models for intervention in hoarding that are already being used in other countries. How does this study represent the next step in knowledge about this topic?**

Thank you for this comment. As outlined above the complex intervention that has been developed from this study is a psychology led multiagency model which although having some similarities to other community partnerships, community response models, community task forces and collaborative agreements, is novel in that it is psychology led. The novel nature of our complex intervention has been highlighted in the conclusion section. In addition, community partnerships, community response models, community task forces and collaborative agreements have not been translated into either UK policy or practice and therefore would need to be investigated in this context which has a very different health and social care system to the USA, Canada and Singapore. We have clarified this further in the introduction. In addition, many of the current models are buried within the grey literature and have not been formally evaluated (this has been highlighted in the text) so our study adds to the emerging body of evidence.

**Why is this type of focus group, with these stakeholders, using the normalization process theory the best path forward to stimulate the creation of the kind of intervention already being used in other countries?**

As stated above and within the manuscript this kind of intervention, while being used in other countries in various forms, has not been translated into either UK policy or practice. In addition, the UK has a very different health and social care system to the USA, Canada and Singapore where most of the other interventions have been developed. Focus groups were chosen as they provided socially negotiated practice examples and key holders were people with a professional interest or concern in hoarding disorder who were involved in some capacity with people with hoarding disorder. NPT influenced these data collection methods, topic guide and data analysis as NPT states that new interventions have the best chance of succeeding if they are based on an awareness and active engagement with existing organisational culture and practices. This has been clarified throughout the manuscript.

**The manuscript does not present much evidence that it relates to hoarding disorder rather than behaviour. Most community-based interventions address hoarding behaviour, as they typically do not assess the psychological factors – such as reasons for saving stuff, whether the stuff was saved intentionally or passively, etc. – that are required to make a diagnosis of hoarding disorder. Quite possibly, the mental health participants in these focus groups would be making hoarding disorder diagnoses, but fire prevention officers and housing officials typically do not conduct assessments that would lead to a diagnosis – such as the reason for accumulating possessions. Is More typically, they’re assessing conditions of the home related to health and safety and adequate maintenance. Without an assessment of the person living in the home (not just conditions of the home), it is not possible to make a diagnosis of any disorder. This was perhaps most evident in the emphasis on the Clutter Scale as a key assessment tool; the Clutter Scale assesses only clutter volume, not any of the reasons how the home came to be the way it is.**

Thank you for this comment. The manuscript focusses on hoarding disorder as the purpose of the psychology led multiagency model would be to allow for diagnosis of hoarding disorder as well as coordination of care based on each patient’s mental stability to cope with intervention. However, as you have rightly identified some of the agencies involved will be dealing with hoarding behaviour prior to any diagnosis however the consensus was that key stakeholders would benefit from a psychology led multiagency model to allow for diagnosis.

**The revised manuscript is much improved in providing scholarly and grey literature citations, but the references do not always support the statements in the text. Sometimes, the text implies an empirical study, whereas the citation is for a review paper or chapter or theoretical paper. For example, line 59 implies that the Bratiotis & Woody paper establishes a heightened risk of squalor in hoarding cases, but it does not. Luu et al. (2018; doi: 10.1016/j.jocrd.2018.08.005), Dong et al. (2012; doi: 10.1177/0898264311425597) and John Snowdon’s work from Australia do establish that elevated risk.**

Thank you for highlighting this. The citation supporting the statement on line 59 has now been changed to Luu M, Lauster N, Bratiotis C, Edsell-Vetter J, Woody SR. Squalor in community-referred hoarded homes. Journal of obsessive-compulsive and related disorders. 2018; 19: 66-71. All other references have been checked for appropriateness.

**Some of the basic information presented about hoarding is not correct. Line 38 implies that hoarding is a synonym for collecting, but it is quite distinct (see Nordsletten et al. 2013, doi: 10.1016/j.comppsych.2012.07.063).**

Thank you for highlighting this. We did not intend to imply that collecting and hoarding were the same thing. We have deleted the reference to collecting on line 38.

**The diagnosis in DSM-5 is called hoarding disorder; compulsive hoarding syndrome was an earlier term that is no longer being used. More broadly, I was confused about the heading, “There is no consensus definition of hoarding disorder.” I think many would argue that DSM-5 presents that definition. Based on the quotations provided, it seems like what the stakeholders were discussing is how to use the word “hoarding” - and I would agree there is no consensus definition for what that means in community settings. What is the threshold for referring to conditions in a home as “hoarding”? The quotes seem to suggest a lack of consensus about that.**

Thank you, we have deleted the sentence which refers to compulsive hoarding syndrome. We have also changed the terminology used throughout the manuscript from “definition” to “understanding” and “prevalence” to “number of cases” so that there is no confusion with the APA/DSM-V definition of hoarding disorder and clinical prevalence.

**I also think it is misleading to suggest that publicity about hoarding, including media reports, increases the prevalence of hoarding disorder. Publicity may result in increased case finding or higher caseloads for stakeholders participating in this study, but it is hard to see how it would result in increased prevalence.**

Thank you for this comment. We have added a sentence in to the introduction regarding how publicity about hoarding has increased the public recognition of hoarding disorder. We have also amended the results to read “increased **identification** of cases was reported to be because of publicity about the disorder and TV documentaries about hoarding disorder”.

**I wondered about the generalizability of the messages in this study. The manuscript states that this is the first study of community-based hoarding intervention practices in the UK. Why is that important? How might the UK’s context differ in relevant ways from the context in other countries (e.g., Canada, US, Australia) where this type of intervention has been going on for awhile? Similarly, the rationale for the study provided in lines 68-70 suggests the importance of translating the work done in other countries to the UK situation but does not articulate how this translation process might be of broader relevance beyond the UK. This generalizability seems important for a journal with an international readership.**

Thank you for highlighting this. We have added to the introduction and the discussion the importance of this research to the UK and how the UK’s context differs in relevant ways from the context in other countries (e.g. Canada, the USA, Australia) where this type of intervention has become institutionalised. We have also discussed the generalisability of our results.

**The Discussion section would be strengthened by being more focused. It presents several interesting points about community-based interventions, but there is no sense of how each one contributes to a larger message.**

Thank you for this suggestion. We have amended the discussion and conclusion sections in order to provide more focus and relevance to our findings. We hope you find these sections improved.

**A more minor question is how was “key” stakeholder defined? What steps were taken to ensure that all the key stakeholder categories relevant to hoarding were represented in the final sample?**

As stated in the manuscript, key stakeholders were defined as people with a professional interest or concern in hoarding disorder. Key stakeholders were identified from and via an existing hoarding research group (https://www.northumbria.ac.uk/about-us/academic-departments/psychology/research/health-and-wellbeing/hoarding-research/), a multidisciplinary group (48 members) which brings together academics from English Universities, stakeholders from the Local Authorities, Housing Associations, Charities, Social Care Services, Mental Health Services, the NHS, and Emergency Services. Many of the key stakeholders were already members of this group, however members were also called upon to identify further key stakeholders. While our sample of stakeholders was relatively small, there was a wide variety of organisations involved; however we have acknowledged as a limitation that there were some services which were not represented such as the ambulance service and the police.
