# Supplementary material for: “In an ideal world that would be a multiagency service because you need everybody’s expertise.” Managing hoarding disorder: A qualitative investigation of existing procedures and practices
Source: PLoS One. 2023 Mar 9;18(3):e0282365. doi: 10.1371/journal.pone.0282365 (PMC9997939; doi:10.1371/journal.pone.0282365)
Supplement: Attachment — Submitted filename: Third response to reviewers.docx [file pone.0282365.s004.docx]

Thank you for taking the time to look at our manuscript again, we have addressed the minor remarks and hope the manuscript is now suitable for publication.

**Reviewer #1: I have no comments. Thank you for addressing all comments and recommendations. Hope your results will contribute to the institutionalization of multidisciplinary teams for HD and the formal evaluation of their work.**

Thank you for all your help improving the manuscript.

**Reviewer #2: Thank you for the invitation to review another version of this manuscript. It is much improved, particularly with regard to the rationale and conceptual underpinnings of the study. The explanation of NPT and how that relates to the study is much clearer now. The various miscommunications that I raised in my previous review have all been addressed. The Discussion section is now clear and succinct.**

Thank you also for your help improving the manuscript.

**I have a few minor remarks that the authors may wish to address.**

**In the first few lines of the Introduction, the language makes it unclear just how the authors define “hoarding”. On line 39, the manuscript states, “humans show a strong tendency to hoard possessions”. The associated reference does not establish this statement as fact (and I suppose it depends on how one defines “hoard”). If this statement is broadly true, then why is the prevalence of hoarding so low? On line 41, the manuscript refers to “normal hoarding tendency”, although what this is remains unclear. I have seen hoarding defined rather broadly as keeping more of something than is needed in the moment (sorry I can’t find the reference). From this perspective, a home pantry or freezer represents hoarding. If this (or something like it) is the meaning of hoarding that the authors intend at the outset of the Introduction, it would be helpful to clarify that because most of the paper is focused on problematic accumulation of possessions.**

Thank you for these comments. The manuscript does indeed focus on hoarding disorder, but we wanted to start by explaining that the accumulation of possession can be a normal human response until it becomes problematic. To clarify this further we have removed the sentence on line 39 which stated that “humans show a strong tendency to hoard possessions” and have amended the wording of the next sentence so that it reads “*accumulating* possessions may be a learnt behaviour, ensuring survival when resources become scare”. In addition we have removed reference to “normal hoarding tendency” so this sentence now reads “However, in a minority of cases (1.5%-2% of the population) saving behaviour becomes excessive and disconnected from any apparent function or purpose, and the person hoards uncontrollably.”

**Lines 71-72 state that “Many of these task forces have become institutionalised within [the US, Canada, and Singapore]”. I’m not sure what it means for a task force to “become institutionalized”, but I’m not sure that has happened in the US and Canada. (I can’t speak about the situation in Singapore.) There are numerous community responses to hoarding in various municipalities, but in practice these interventions have precarious funding and remain difficult to access. [As an aside, and as demonstration that the manuscript has ignited my interest and curiosity, I am aware of very few formal policies related to hoarding (the few examples that I can recall are limited to housing providers), although of course formal policies would be very helpful. Another difference between the situation in the US and Canada and what I think you’re proposing for the UK is that in the US and Canada, these community-based approaches are typically approached from the perspective of tenancy preservation or fire prevention rather than as health care.]**

Thank you for this useful comment, we have removed the sentence suggesting community task forces have become institutionalised.

**Line 259 makes reference to the Clutter Scale. I think this is referring to the Clutter Image Rating scale. If that’s correct, it would probably be better to use the full name so interested readers can more accurately access it online.**

Thank you for this suggestion, we are indeed referring to the Clutter Image Rating Scale, so we have used the full name on line 259 as well as in the abstract.
